# Special Issue “Bioceramics: Challenges and Medical Applications of Calcium-Phosphate-Based Biocompatible Ceramics”

**DOI:** 10.3390/ijms262110612

**Published:** 2025-10-31

**Authors:** Kazuaki Hashimoto, Mamoru Aizawa

**Affiliations:** 1 Department of Applied Chemistry, Faculty of Engineering, Chiba Institute of Technology, 2-17-1 Tsudanuma, Narashino 275-0016, Chiba, Japan; 2 Department of Applied Chemistry, School of Science and Technology, Meiji University, 1-1-1 Higashimita, Tama-ku, Kawasaki 214-8571, Kanagawa, Japan; mamorua@meiji.ac.jp

Calcium-phosphate (CaP) ceramics have long occupied a central role in biomaterials science owing to their chemical similarity to bone mineral and favorable biological performance [1,2,3]. Early work by de Groot on hydroxyapatite coatings [4] and Hench’s introduction of bioactive glasses [5] established the foundation for modern bioceramics. Over subsequent decades, CaP research expanded beyond hydroxyapatite (HAp), octacalcium phosphate (OCP), and β-tricalcium phosphate (β-TCP) to encompass hybrid composites, ion substitution strategies, and surface functionalization techniques [6,7,8,9].

## 1. Hydroxyapatite and Related Phosphates

Hydroxyapatite remains the archetype of bone substitute ceramics due to its stability and osteoconductivity [10,11]. Silicon-substituted HAp has demonstrated enhanced bioactivity [12], while zinc- and strontium-doped systems show improved osteogenesis [13,14]. Beyond composition, Gibson and Bonfield’s studies highlighted the role of trace substitution in tailoring HAp reactivity [15]. Contemporary approaches integrate mesoporous structures [16] and composite scaffolds [17] to facilitate drug delivery and vascularization.

## 2. Octacalcium Phosphate and Osteogenesis

OCP has emerged as a key transient phase with high osteoinductive potential [18]. Its transformation into HAp under physiological conditions supports new bone formation. Pioneering studies by Suzuki and colleagues revealed protein adsorption effects on OCP surfaces [19], while Wang et al. demonstrated enhanced osteogenesis in OCP–polymer composites [20]. Parallel research in *Biomaterials* and *Acta Biomater* confirmed OCP’s clinical promise [21,22].

## 3. β-Tricalcium Phosphate and Functionalization

β-TCP exhibits higher resorbability than HAp, making it an ideal candidate for resorbable scaffolds [23,24]. Wu and Chang demonstrated structure–function relationships in porous β-TCP [25], and Hashimoto et al. reported freeze-dried β-TCP scaffolds with tunable porosity [26]. Electrical polarization, as pioneered by Yamashita et al. [27], modulates cellular responses, a finding later confirmed in polarized β-TCP [28]. Recent work has further expanded to β-TCP doped with Mn, Mg, and Si, demonstrating improved mechanical reliability and bioactivity [29,30].

## 4. Hybrid Composites and Interfacial Engineering

Hybrid architectures combining CaPs with polymers, metals, and bioactive molecules enable multifunctional performance [31,32,33,34,35,36]. Vallet-Regí introduced mesoporous silica for drug delivery, further extending CaP applications [37]. Advances in additive manufacturing allow precise 3D-printed CaP composites with hierarchical porosity [38]. These approaches highlight the critical role of interfacial chemistry and microstructural design.

## 5. Nanostructures, Antibacterial Strategies, and Metals

Nanostructural control is central to regulating bioactivity and degradation kinetics [39]. Lactoferrin-functionalized CaP nanoparticles demonstrated antibacterial potential [40]. Metallic systems, such as bioactive Ti and Zr alloys treated in simulated body fluid [41], represent hybrid strategies bridging ceramics and metals. Antibacterial coatings and silver- or copper-doped CaPs further broaden biomedical applications [42].

## 6. Future Outlook

The future of CaP bioceramics will rely on integrating traditional materials science with emerging technologies. Machine learning and AI-driven design [43,44,45], self-driving laboratories [46], and predictive modeling of bioactivity [47] are accelerating discovery. Clinically, the growing burden of osteoporosis and musculoskeletal disease underscores the need for biomaterials that combine osteoconductivity, resorbability, antibacterial function, and structural integrity [48,49,50]. Progress will depend on multidisciplinary collaboration among chemists, biologists, engineers, and clinicians.
